# The Utility of Intraoperative Microbiological, Cytological, and Histopathological Sampling in the Setting of an Empyema Necessitating Lung Decortication Surgery

**DOI:** 10.7759/cureus.8839

**Published:** 2020-06-26

**Authors:** Muhammad Ali Niaz, Talal Almas, Leo Phelan, Maryam Ehtesham, David G Healy

**Affiliations:** 1 Surgery, Royal College of Surgeons in Ireland, Dublin, IRL; 2 Internal Medicine, Royal College of Surgeons in Ireland, Dublin, IRL; 3 Thoracic Surgery, St. Vincent's University Hospital, Dublin, IRL

**Keywords:** empyema, decortication

## Abstract

Introduction and Aims: Empyema thoracis is a pleural space pathology that indicates accumulation of purulent material in the pleural space. It is often associated with an underlying infectious process, such as pneumonia, but can also be a ramification of a more sinister etiology, such as lung carcinoma, often warranting lung decortication surgery for prompt resolution. Although radiological imaging is used to form a preliminary diagnosis, its true predictive value remains questionable, and intraoperative microbiological, cytological, and histopathological samples are thus instrumental in yielding helpful diagnostic information. This study aims to gauge whether intraoperative microbiological, cytological, and histopathological analyses yield any additional diagnostic information in establishing the etiology underlying empyema, necessitating decortication surgery.

Methods: Microbiological, cytological, and histopathological records of 43 patients undergoing decortication surgery were included in this study. Only patients who were diagnosed with late stages of empyema and subsequently underwent decortication surgery were included in this study.

Results: The sample consisted of 43 patients, including 23 males and 20 females. For microbiology, 4.88% of the bronchoalveolar lavage (BAL) samples, 7.69% of tissue fluid samples, and 7.32% of pleural fluid samples were positive for an infectious microorganism. For cytology/histopathology, 0.00% of BAL samples, 5.41% of pleural fluid samples, and 7.32% of tissue samples were positive for an underlying infective etiology.

Conclusion: For the study and analysis of the microbiological samples, a myriad of all three different modalities of diagnosis is essential. However, tissue sampling is the preferred modality of diagnosis for cytology/histopathology owing to its ability to detect positive cases that might otherwise evade prompt detection.

## Introduction

Empyema, also called purulent pleuritis or pyothorax, is a pathology that evokes the accumulation of purulent material in the area between the lungs and the inner chest wall, known as the pleural space [1]. It most frequently occurs as long-term sequelae of pneumonia but is also considered to be associated with lung carcinoma, diabetes, immunosuppression, pleural effusion, and even with necrotic tissue following invasive medical procedures [2]. Empyema often presents with distinct respiratory symptoms, but can also present with insidious onset of non-specific clinical features, obscuring the diagnosis further [3]. These symptoms may include lethargy, fever, night sweats, and cough productive of purulent sputum. The perplexing clinical presentation and variable etiologies of empyema make it a diagnostic challenge for clinicians [4].

The pathogenesis of empyema involves three stages, which initially involve an excessive build-up of pleural fluid in the pleural cavity, an acidic microenvironment (pH greater than or equal to 7.2), and lactate dehydrogenase levels below 1,000 IU/L [5]. Inappropriate management of this first stage can result in an exacerbated second (fibrinopurulent) stage that is characterized by a dense exudate on a background of fibrin [6]. Unresolved fibrinopurulent stage entails the exorbitant proliferation of fibroblasts, ultimately leading to a tertiary (organizing) stage that produces peels of inelastic nature [7]. These peels eventually besiege the lungs and pulmonary circuit leading to restricted lung movements, thereby necessitating prompt invasive action by decortication surgery [8]. Therefore, an accurate and timely diagnosis is vital before it becomes challenging to treat.

Management of empyema varies vastly, and radiological imaging techniques are often used in the preliminary diagnosis of empyema, but due to the poor prognostic value, their utility in yielding a definitive cause of empyema remains limited [9]. If the pleural infection is identified, prompt commencement of an antimicrobial regimen is required [10]. In many cases, empyema is detected at late stages which require surgical intervention [11]. To determine the etiology, prognosis, and treatment underlying suppuration and empyema, various techniques, such as bronchoalveolar lavage (BAL), pleural fluid sampling, and tissue sampling, are routinely employed [1,4,11]. In many cases, the patients referred to surgery are already undergoing antimicrobial therapy so the results from these tests might often be unrewarding.

BAL is used as a diagnostic tool for empyema. The advent of modern diagnostic modalities has rendered BAL a relatively safe procedure, and one that is widely used in clinical settings due to its non-invasive nature [1,7]. However, samples obtained from BAL often prove invaluable in yielding information about the dysplastic nature of cells and any underlying infectious ailment. Similarly, pleural fluid analysis is used to guide the management and is recommended for patients suffering from pneumonic illness and ongoing sepsis who have a subsequent pleural effusion with a depth >10 mm [6,11]. The relative ease of access to the percutaneous pleural space for tapping pleural effusion leaves the procedure liable to various complications [12]. Image guiding, such as ultrasound, can be used to minimize the risk of organ perforation and reduce patient discomfort [13]. Microbiology, such as culture and gram stain, is useful in determining the particular bacteria associated with infection and thus aid in an antibiotic prescription [14]. Cytology of pleural fluid is a very efficacious method to diagnose malignant pleural effusion and remains vital in ruling out any possibility of malignancy as a predisposing factor for empyema. 

Thoracoscopy is an invasive procedure diagnostic and therapeutic procedure usually employed while decortication surgery is underway in patients with late-stage pleural empyema [9,15]. Although less invasive diagnostic modalities are available to diagnose the underlying cause of empyema, diagnostic thoracoscopy is performed due to its ability to yield definitive diagnoses and high sensitivity rates [15]. The objective of this study is to discern whether intraoperative microbiological, cytological, and histopathological analyses yield any additional diagnostic information pertaining to the etiology underlying empyema thoracis. 

## Materials and methods

In this retrospective review, prospectively recorded data of patients undergoing decortication surgery for empyema at St. Vincent’s University Hospital, Dublin were ascertained. Microbiological and histopathological records of BAL, pleural fluid, and tissue samples were obtained from January 2011 to June 2019. A total of 43 patients were included in this review. Only patients who were diagnosed with late stages of empyema and underwent decortication surgery were included in this study. Patients who had been diagnosed with mesothelioma, lung carcinoma, or any other kind of pleural pathology were excluded. The study solely focused on samples taken intraoperatively for microbiological analysis, cytology, and histopathology. The data were obtained from BAL, pleural fluid, and decorticated material (pleural/lung tissue). The BAL was obtained with flexible bronchoscopy. Approximately 100 ml of 0.9%, saline water was instilled in a subsegment of each lung and was sucked back. The lavage obtained was then stored and sent to the lab in different containers under sterile conditions to prevent contamination. Microbiological examination, including gram stain and culture, was performed. Additionally, cytological examination of the lavages was also performed. Pleural fluid samples were then taken from the affected side of pleural space and were sent to the lab for microbiological examination, including culture and gram stain. Furthermore, cytological examination of the pleural fluid samples was carried out. Tissue samples from decorticated material during the surgery were collected under sterile conditions. Gram stain, culture, enrichment culture, and extended incubation were carried out on all the samples. Finally, histological tests were performed on the collected samples.

## Results

The sample consisted of 43 patients; 23 (53.5%) males and 20 (46.5%) females were included in the study. The mean age of patients was 51 years. The numbers of patients afflicted with a right-sided empyema and a left-sided empyema were 22 and 21, respectively. Only one patient was suffering from both sided empyema. The results can broadly be divided with regards to the different modalities of diagnosis that were used. The distribution of patients concerning the site of empyema is tabulated in Table 1.

**Table 1:** The distribution of patients with pertinence to the various sites of empyema.

Number of patients	43
Male	23
Female	20
Left lung empyema	20
Right lung empyema	22
Both lung empyema	1

**Figure 1:**
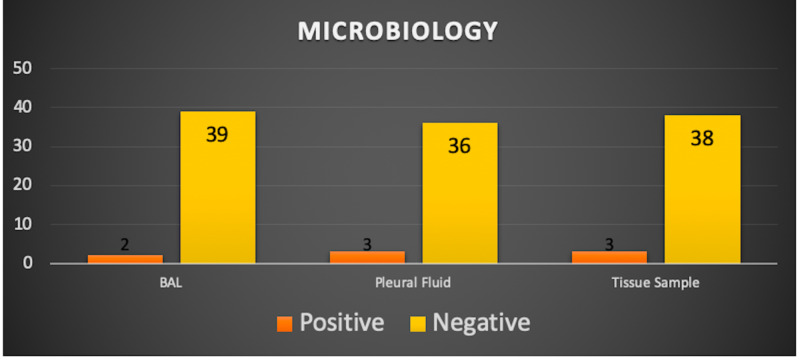
The microbiology results as they relate to the different modalities of diagnosis employed.

Furthermore, the results obtained from the different modalities (microbiology, cytology, and histopathology) of diagnoses, such as BAL, pleural fluid samples, and tissue samples, are elucidated (Figure 1). 

**Figure 2:**
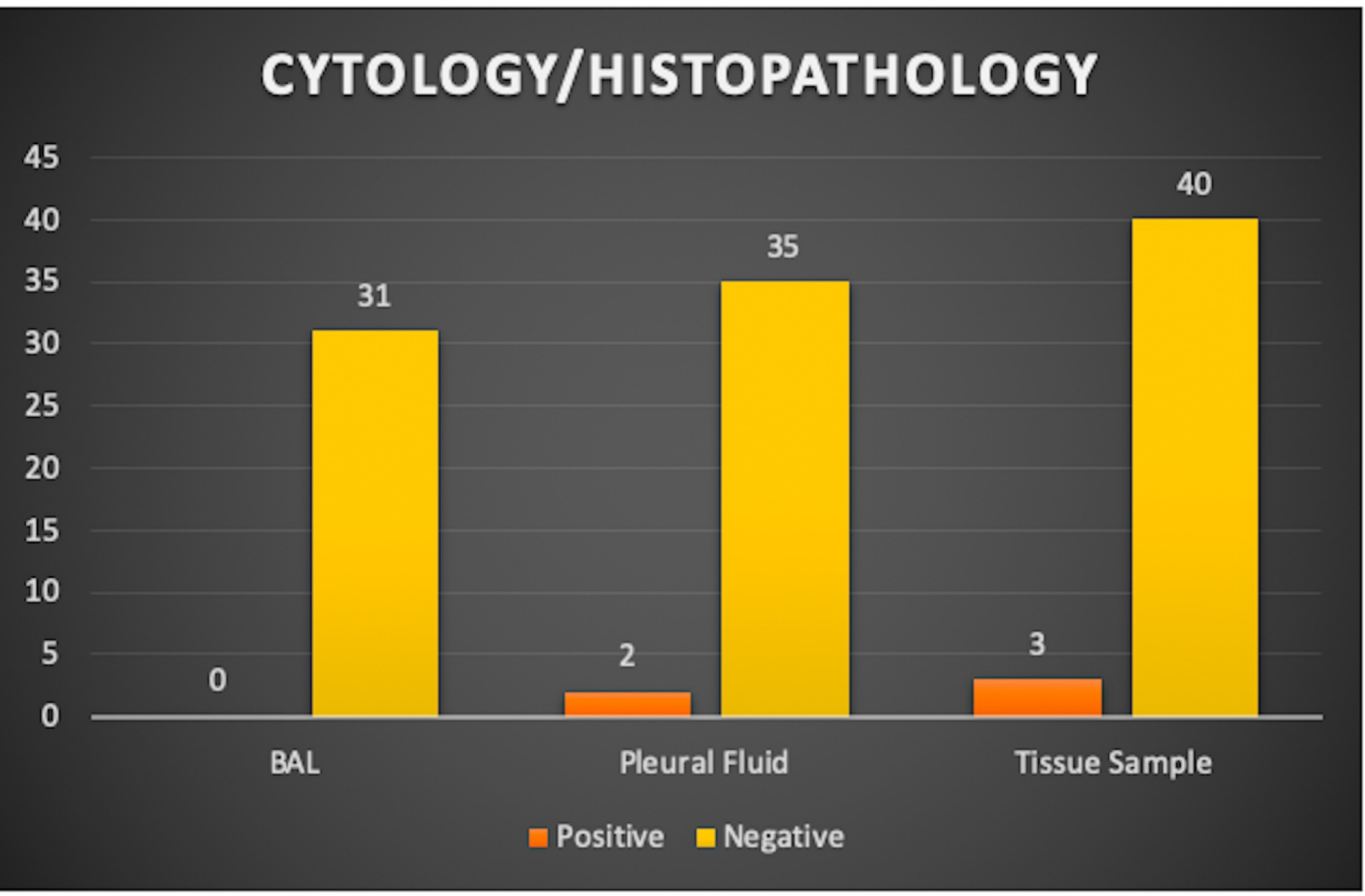
The cytology/histopathology results as they relate to the different modalities of diagnosis.

As it can be noted, a vast majority of the results obtained turned out to be negative, owing primarily to the fact that patients who are referred to tertiary care hospitals for further referral and surgery tend to be adherent to an antimicrobial regimen before surgery. This regimen, in turn, effectively combats the underlying pathogenic causes, resulting in largely negative results upon intraoperative sampling. Figure 2 delineates the results obtained from the cytology/histopathology.

It is easily discerned that BAL detected the least number of positive cases, while pleural fluid and tissue samples yielded the same numbers of positive results. 

## Discussion

Empyema thoracis is a potentially grave pathology that can afflict the pleural space by infectious ailments, such as pneumonia and tuberculosis. Of the plethora of long-term sequelae associated with these infectious ailments, one is empyema thoracis [4,15]. Despite the advent of revolutionary medical practices and therapeutic regimens, the mortality rate associated with empyema thoracis remains intimidatingly high, hovering around 24% [16]. The high morbidity and mortality associated with empyema thoracis indicate the need for evaluation of the efficacy of the intraoperative sampling techniques as a useful resource in the management of the disease. 

The major drawback of relying on procedures such as BAL as a diagnostic modality for empyema is the vast range of normal values and dilution factors that undermine its comparability [17]. BAL cytology can be used to diagnose malignancies, which often predispose patients to the development of empyema [18]. In a study by Bezel et al., BAL’s sensitivity for the diagnosis of carcinoma was found to be 29% with an overall diagnostic yield of 46%, thus proving its meager diagnostic ability in the diagnosis of carcinoma [17]. The low sensitivity and diagnostic yield of BAL suggest the need for its assessment as a diagnostic investigation for empyema.

In diagnosing malignant pleural effusion, if the previous investigation turns negative, thoracoscopy is considered to be the gold standard due to its high diagnostic yield, ranging from 93% to 97% [14]. Histological and microbiological tests of tissue samples, such as culture and gram stain, are performed to ascertain the underlying cause(s) of empyema [15]. Previous studies have shown the very high diagnostic sensitivity of thoracoscopy to be as high as 95% in malignancy and 100% in benign diseases [14,19,20]. This shows the value of thoracoscopy in managing complicated cases of empyema that require surgical intervention.

Initial management of empyema thoracis revolves primarily around the uptake of a concoction of antibiotics and antivirals to thwart superimposed infection [21]. In cases that have progressed and are thus not amenable to conservative management, lung decortication surgery remains the last viable option [2,4,21]. Before the decortication surgery, a myriad of antibiotics is instituted, which means that intraoperative samples often yield false-negative culture results [3,22]. One study suggests that detailed evaluation using various samples, such as BAL, pleural fluid, and tissue samples, is performed to establish the cause underlying the empyema. The discussion below elucidates the clinical utility of microbiology, cytology, and histopathology as it pertains to these various aforesaid samples. 

Microbiology

Upon analysis, it appears that while all three modalities afford various diagnostic advantages, tissue samples and pleural fluid samples emerge as the clear modalities of choice. We note from Figure 1 that BAL detected two cases that were positive compared to tissue samples and pleural fluid samples, which detected three positive cases each. Regardless, none of the aforementioned modalities can be regarded as futile. Similar conclusions are driven by a myriad of studies [13,23]. We also observe that all the different modalities detected positive cases in patients in which the other modalities failed to yield positive results. For instance, a patient whose BAL sample turned out to be positive did not, contrary to expectation, also have positive tissue samples and pleural fluid, and without BAL, these cases might have been misdiagnosed as false negatives. 

Cytology

Cytology results, when evaluated in terms of the different modalities, divulged a different scenario. For instance, the BAL results obtained from cytology reinforce the fact that the utility of BAL remains questionable; BAL remained unsuccessful in detecting any positive cases. On the other hand, tissue samples remained successful in detecting three positive cases, of which only two were also additionally detected by the pleural fluid cytology. Consequently, it can also be extrapolated that the two positive cases detected by pleural fluid were also indeed detected by tissue sampling. This leads us to the riveting conclusion that tissue sampling is the most invaluable diagnostic modality from amongst the ones studied. A recent study conducted by Ferguson et al. highlighted the limited role of cytology in diagnosing parapneumonic effusions and thoracic empyema [24]. Nevertheless, the diagnostic yield of pleural fluid cytology from previous studies ranges from 40% to 87% and depends largely on a plethora of factors, such as the extent of disease, nature of primary malignancy, and the histological type of malignancy [12,23]. A combination of cytology with the other modalities of diagnosis should therefore be employed to evaluate the etiology underlying empyema thoracis, which can in turn necessitate lung decortication surgery [25]. 

The abstract of this article has been presented in the Irish Thoracic Society Annual Scientific Meeting 2019 [25].

## Conclusions

Evaluation of intraoperative samples to establish the etiology underlying empyema is pivotal. Although the employment of a concoction of diagnostic modalities can often pose financial challenges to tertiary hospitals, the synergy of these modalities provides additional diagnostic information on the etiology underlying the empyema. For the study and analysis of the microbiological and cytological/histopathological samples, a slate of all three different modalities of diagnosis is essential. Contrarily, for the analysis of cytology/histology, only tissue sample is the preferred modality of diagnosis, owing to its ability to detect positive cases that cannot otherwise be detected by the remainder of the modalities studied. 

## References

[1] Acharya PR, Shah KV (2007). Empyema thoracis: a clinical study. Ann Thorac Med.

[2] Froeschle P, Wanke W, Granetzny A (2005). Video-thoracoscopy and staged management of preoperative empyema in lung cancer. Thorac Cardiovasc Surg.

[3] Casal RF, Eapen GA, Morice RC, Jimenez CA (2009). Medical thoracoscopy. Curr Opin Pulm Med.

[4] Nadeem A, Bilal A, Shahkar S, Shah A (2004). Presentation and management of empyema thoracis at Lady Reading Hospital Peshawar. J Ayub Med Coll Abbottabad.

[5] Hajjar WM, Ahmed I, Al-Nassar SA, Alsultan RK, Alwgait WA, Alkhalaf HH, Bisht SC (2016). Video-assisted thoracoscopic decortication for the management of late stage pleural empyema, is it feasible?. Ann Thorac Med.

[6] Elsayed HH, Mostafa A, Fathy E (2018). Thoracoscopic management of early stages of empyema: is this the golden standard?. J Vis Surg.

[7] Davies HE, Davies RJ, Davies CW, BTS Pleural Disease Guideline Group (2010). Management of pleural infection in adults: British Thoracic Society Pleural Disease Guideline 2010. Thorax.

[8] Radha S, Afroz T, Prasad S, Ravindra N (2014). Diagnostic utility of bronchoalveolar lavage. J Cytol.

[9] Vaziri M, Abed O (2012). Management of thoracic empyema: review of 112 cases. Acta Med Iran.

[10] Abbas N, Tolan M, Healy D (2015). Are operative microbiology samples warranted in the setting of a stage III empyema thoracis?. J Cardiothorac Surg.

[11] Molnar TF (2007). Current surgical treatment of thoracic empyema in adults. Eur J Cardiothorac Surg.

[12] Lamont T, Surkitt-Parr M, Scarpello J, Durand M, Hooper C, Maskell N (2009). Insertion of chest drains: summary of a safety report from the National Patient Safety Agency. BMJ.

[13] Rosenstengel A (2012). Pleural infection: current diagnosis and management. J Thorac Dis.

[14] Dixit R, Agarwal KC, Gokhroo A, Patil CB, Meena M, Shah NS, Arora P (2017). Diagnosis and management options in malignant pleural effusions. Lung India.

[15] Bollmann BA, Seeliger B, Drick N, Welte T, Gottlieb JT, Greer M (2017). Cellular analysis in bronchoalveolar lavage: inherent limitations of current standard procedure. Eur Respir J.

[16] Harris RJ, Kavuru MS, Mehta AC, Medendorp SV, Wiedemann HP, Kirby TJ, Rice TW (1995). The impact of thoracoscopy on the management of pleural disease. Chest.

[17] Bezel P, Tischler V, Robinson C (2016). Diagnostic value of bronchoalveolar lavage for diagnosis of suspected peripheral lung cancer. Clin Lung Cancer.

[18] Urso B, Michaels S (2016). Differentiation of lung cancer, empyema, and abscess through the investigation of a dry cough. Cureus.

[19] Biswas B, Sharma SK, Negi RS, Gupta N, Jaswal VM, Niranjan N (2016). Pleural effusion: role of pleural fluid cytology, adenosine deaminase level, and pleural biopsy in diagnosis. J Cytol.

[20] Ahmed AE, Yacoub TE (2010). Empyema thoracis. Clin Med Insights Circ Respir Pulm Med.

[21] Sumalani KK, Rizvi NA, Asghar A (2018). Role of medical thoracoscopy in the management of multiloculated empyema. BMC Pulm Med.

[22] Ohara G, Tamura T, Satoh H (2016). Decortication of empyema. Ann Thorac Med.

[23] Walters J, Foley N, Molyneux M (2011). Pus in the thorax: management of empyema and lung abscess. Contin Educ Anaesth Crit Care Pain.

[24] Ferguson J, Kazimir M, Gailey M, Moore F, Schott E (2020). Predictive value of pleural cytology in the diagnosis of complicated parapneumonic effusions and empyema thoracis. Pulm Med.

[25] Niaz MA, Phelan L, Healy DG (2019). The utility of intra-operative microbiological, cytological and histopathological sampling in the lung decortication surgery. Ir J Med Sci.

